# Antibodies get under the skin

**DOI:** 10.7554/eLife.104040

**Published:** 2024-10-30

**Authors:** Chiara Levra Levron, Gabriele Piacenti, Giacomo Donati

**Affiliations:** 1 Department of Life Sciences and Systems Biology and the Molecular Biotechnology Center “Guido Tarone”, University of Turin Torino Italy

**Keywords:** notch signaling, therapeutic antibodies, cell fates, differentiation, stem cell, Mouse

## Abstract

By inhibiting receptor-ligand interactions in sebaceous glands, antibodies may be able to treat certain skin conditions.

**Related research article** Abidi SNF, Chan S, Seidel K, Lafkas D, Vermeulen L, Peale F, Siebel CW. 2024. The Jag2/Notch1 signaling axis promotes sebaceous gland differentiation and controls progenitor proliferation. *eLife* **13**:RP98747. doi: 10.7554/eLife.98747.

As the outer organ of the body, the skin has many roles, from defending against microbes to regulating body temperature and sensations. To exercise these duties, it takes advantage of various types of cells, tissues and glands. Sebaceous glands – which are found in hair follicles all over the body, apart from the palms of our hands and the soles of our feet – produce a lipid-rich liquid called sebum, which prevents the skin from becoming too dry. Sebum also helps protect against bacterial and fungal infections, and can have pro- and anti- inflammatory effects.

The cells that produce sebum are called sebocytes, and they undergo a process of differentiation that starts with stem cells in the peripheral zone, followed by a period in the maturation zone (where the sebocytes accumulate lipids and become enlarged), before they move to the degeneration zone and burst, releasing sebum into the sebaceous duct, which carries it to the skin surface (Figure 1A; Geueke and Niemann, 2021; Oulès et al., 2020). Defective sebaceous glands have been linked to numerous skin conditions, including alopecia, acne vulgaris, psoriasis and seborrheic dermatitis, as well as tumors such as sebaceoma, sebaceous adenoma and carcinoma (Oulès et al., 2019). Therefore, there is a clear need for a better understanding of the molecular mechanisms underpinning the development and workings of the sebaceous glands.

**Figure 1.**
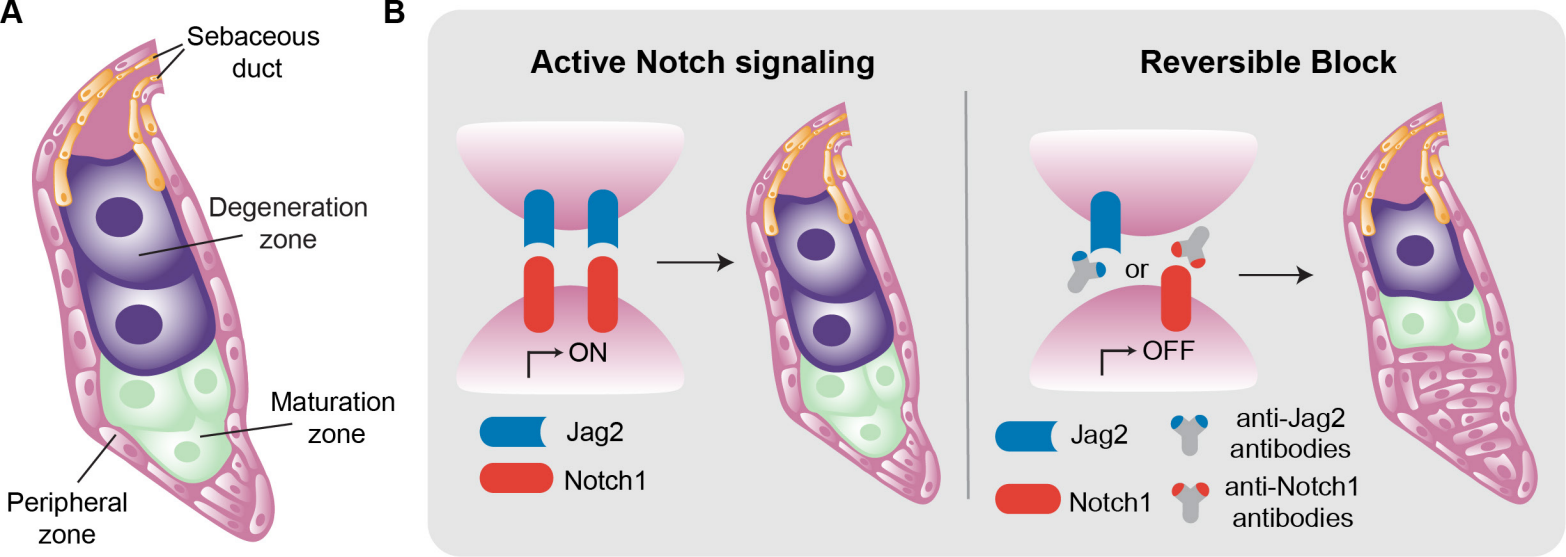
Inside the sebaceous gland. (**A**) Schematic representation of a sebaceous gland. Stem cells (pink) in the peripheral zone differentiate to become sebocytes (green) as they move into the maturation zone. The process of differentiation continues, and the fully differentiated cells (purple) then move into the degeneration zone, where they accumulate lipids and increase in size. Eventually the sebocytes burst and the lipid-filled sebum travels to the skin via the sebaceous duct (orange). (**B**) The Notch signaling pathway is activated when the ligand Jag2 (blue) binds to the Notch1 receptor (red). This pathway drives the process of differentiation in the sebaceous gland, from stem cells to mature sebocytes (left). The interaction between Jag2 and Notch1 can be blocked with anti-Jag2 antibodies (grey and blue Y-shape) or anti-Notch1 antibodies (grey and red Y-shape). This, in turn, blocks sebocyte differentiation, leading to an increase in the number of stem cells and a reduction in the number of mature sebocytes (right).

The Notch signaling pathway is a key regulator of stem cell differentiation and is known to impact sebaceous glands. The pathway is activated when transmembrane Notch receptors bind to their corresponding ligands on the surface of sebocytes. Mammals express four Notch receptors (called Notch1, 2, 3, and 4), and five ligands have been described (Jag1 and 2, and Delta-like1, 3 and 4; Nowell and Radtke, 2013; Zhou et al., 2022). Previous work has shown that deleting the gene for Notch2 in mice does not affect sebaceous glands (Pan et al., 2004). Other research has shown that genetically ablating Notch1 in adult mice suppresses the differentiation of sebocytes (Veniaminova et al., 2019), but the identity of the ligand required to activate the pathway remained a mystery. Now, in eLife, Syeda Nayab Fatima Abidi, Christian Siebel and colleagues at Genentech report using monoclonal antibodies to show that Notch1 and Jag2 ligand are required for sebocyte differentiation in mice (Abidi et al., 2024).

First, using two different monoclonal antibodies that bind specifically to and inhibit either Notch1 or 2 (Lafkas et al., 2015), Abidi et al. showed that only inhibiting Notch1 reduced the differentiation of the cells in sebaceous glands, confirming its primary role in regulating sebocyte fate. Next, the team focused on identifying the Notch ligands involved. Experiments with two specific antibodies against Jag1 and Jag2 revealed that blocking Jag1 did not affect sebocyte differentiation. However, when Jag2 was blocked, very few mature sebocytes were produced, and the gland was mostly filled with immature proliferating cells that had not differentiated (Figure 1B).

Since the antibodies used to inhibit Notch1 and Jag2 are eventually cleared from the system, the researchers were able to study if the transient inhibition of Notch1-Jag2 binding had permanent consequences. They found that, after antibody clearance, Notch activity was re-established and sebocyte differentiation also recovered, revealing that the transient Notch1-Jag2 inhibition had only temporary impacts on the differentiation process, without limiting the potential of the stem cells in the gland. Furthermore, these antibodies seemed to specifically affect the sebaceous glands and had little or no impact on other components of the skin such as the adipocytes and the interfollicular epidermis.

The value of monoclonal antibodies from a clinical perspective is evident: they show remarkable target specificity and numerous antibody therapies have already been approved for clinical use (Shepard et al., 2017). The reversibility of the effects induced by anti-Jag2 antibodies provides translational potential for disorders involving sebocyte overactivity, such as acne. These antibodies also have the advantage that they seem to only affect the differentiation of sebaceous glands, with negligible effects on other cellular components of the skin.
